# JR20, a novel natural product-derived compound, exhibits potent anti-biofilm activity against methicillin-resistant *Staphylococcus aureus*

**DOI:** 10.3389/fmicb.2025.1743534

**Published:** 2026-01-22

**Authors:** Meirong Zhao, Chaowei Zhang, Yogini Jaiswal, Xinrong Xie, Dongyu Huang, Zhendan He, Leonard Williams, Yifu Guan, Hedong Bian, Xun Song

**Affiliations:** 1 School of Chemistry and Chemical Engineering, Guangxi Minzu University, Nanning, China; 2 College of Food and Pharmaceutical Engineering, Guangxi Vocational University of Agriculture, Nanning, China; 3 College of Pharmacy, Shenzhen Technology University, Shenzhen, China; 4 School of Chinese Medicine, Hong Kong Baptist University, Kowloon Tong, Hong Kong, China; 5 Center for Excellence in Post-Harvest Technologies, North Carolina Agricultural and Technical State University, The North Carolina Research Campus, Kannapolis, NC, United States; 6 School of Pharmacy, Shenzhen University Medical School, Shenzhen University, Shenzhen, China

**Keywords:** antibiotic-resistant bacteria, arylnaphthalene lignan, biofilm disruption, confocal microscopy, MRSA

## Abstract

**Objective:**

JR20, a novel sesamin-derived arylnaphthalene lignan, has demonstrated potent antifungal activity. This study further investigates its antibacterial potential against MRSA (methicillin-resistant *Staphylococcus aureus*).

**Methods:**

The highlights of this research include the use of SYTO9 and PI fluorescence double staining, along with three-dimensional confocal microscopy to reveal the thickness and viability of biofilms under JR0′s influence. Additionally, scanning and transmission electron microscopy were employed to observe the morphological changes of MRSA under JR0′s impact. By combining the observed reduction in ATP content within MRSA, a preliminary mechanism was hypothesized. *In vivo* anti-infection experiments were further conducted to evaluate the compound's biological activity in liver and spleen tissues of mice.

**Results:**

JR20 exhibited potent anti-MRSA activity (IC_50_ = 20.88 μg/mL). Mechanistic investigations revealed multi-level effects: confocal microscopy demonstrated altered biofilm thickness and viability; SEM/TEM confirmed distinct morphological changes in bacterial cells; And ATP content reduction indicated metabolic disruption. *In vivo* experiments validated these antibacterial effects and further revealed anti-inflammatory properties, underscoring JR0′s therapeutic potential against MRSA infections.

**Conclusion:**

This study confirms JR0′s potent anti-MRSA activity, clarifies its effects on biofilms and MRSA morphology, and proposes a preliminary mechanism by reduced ATP. JR20 demonstrates significant potential for combating drug-resistant bacteria and advancing antibiofilm drug discovery.

## 1 Introduction

Antimicrobial-resistant bacteria have long been regarded as a persistent threat to human health. According to the Centers for Disease Control and Prevention's (CDC) Antimicrobial Resistance Threat Report, over 2.8 million cases of antimicrobial-resistant infections occurred annually in the United States in 2019. These resulted in more than 35,000 deaths. Hospital-acquired infections showed no signs of reduction through 2022, with an increase of 20% during the COVID-19 pandemic. Among these, methicillin-resistant *Staphylococcus aureus* (MRSA) is classified as a severe threat (Prevention, 2019, 2025). After obtaining the heterologous penicillin-binding protein 2a (PBP2a) encoded by the mecA gene, the N-terminal non-penicillin-binding domain (residues 27-326) of the protein interacts with the C-terminal transpeptidase domain (residues 327-668), through conformational coupling. Among them, the key residues Tyr446 and Met641 act as “gate switches”. They hinder drug binding in the closed conformation, resulting in a significant decrease in the sensitivity of their active center Ser403 to penicillin. As a result, MRSA gained the ability to escape the bactericidal effects of penicillin-resistant beta-lactam antibiotics, especially methicillin (Hartman and Tomasz, 1984; Matsuhashi et al., 1986).

Furthermore, MRSA acts as a common precursor to biofilms because of its capacity to colonize mucosal surfaces or organs. Biofilms composed of proteins and polysaccharides can adhere to both organic and inorganic surfaces, leading to various chronic illnesses in humans. The production of this substance contributes to the challenge of treating MRSA resistance (Kaushik et al., 2024; Yu et al., 2024; Morrison et al., 2015). Currently, Vancomycin (VAN) is the primary drug used to treat infections caused by this bacterium, as it inhibits bacterial cell wall synthesis to exert its bactericidal effect. It exhibits good antibacterial activity against many Gram-positive bacteria, including MRSA. However, long-term misuse has led to the emergence of vancomycin-resistant *Enterococcus* (VRE) and even vancomycin-resistant *Staphylococcus aureus* (VRSA), thereby limiting its clinical application (Hiramatsu et al., 1997; Tenover et al., 2001; Lakhundi and Zhang, 2018). Given the extent of its resistance evolution and persistently high rates of hospital-acquired infections, there is an urgent need to develop novel antimicrobial agents effective against multidrug-resistant bacteria, such as MRSA (Jimenez et al., 2012; Deurenberg and Stobberingh, 2008).

JR20 (Exact Mass: 350.0790) is a novel arylnaphthalene lignan, synthesized from the natural product sesamin. Based on previous antimicrobial screening results, JR20 has been characterized as a compound with broad-spectrum antimicrobial activity. The synthetic route and fundamental studies related to JR20 have been documented in earlier publications (Wu et al., 2024; Zhang et al., 2025). This study included several phenotypic assays to evaluate the anti-MRSA activity of JR20. The findings showed that JR20 has strong bactericidal effects, particularly against the formation of biofilms, in addition to its inhibitory properties. According to these results, JR20 functions as a strong antibacterial agent that is highly effective against biofilms, which are a key component of MRSA pathogenicity.

## 2 Materials and methods

### 2.1 Strain and reagents

All chemical reagents and biological materials used in this study were obtained from commercial suppliers with specified purity grades. The methicillin-resistant *Staphylococcus aureus* strain (ATCC 43300) was acquired from the American Type Culture Collection (ATCC, USA). The strain was cultured in Tryptic Soy Broth (TSB) liquid medium and on Tryptic Soy Agar (TSA) solid medium. Both media along with vancomycin HCl (VAN) and crystal violet, were procured from Dalian Meilun Biotechnology Co., Ltd (Dalian, China), which also supplied the dimethyl sulfoxide (DMSO). CellTiter-Glo^®^ 2.0 was purchased from Promega Biotechnology Co., Ltd (Beijing, China). LIVE/DEAD™ BacLight™ Bacterial Viability Assay Kit (L7007) was purchased from Thermo Fisher Scientific Co., Ltd (Shanghai, China). Glutaraldehyde 2.5% (EM grade) was purchased from Beijing Solarbio Science & Technology Co., Ltd (Beijing, China). All other organic solvents including ethanol, were of analytical grade.

### 2.2 Synthesis of arylnaphthalene lignan JR20

#### 2.2.1 Synthesis of compound 2

To a stirred solution of sesamin (1.1 g, 3.0 mmol) in CH_2_Cl_2_ (20 mL), acetic anhydride (5 mL) was added. The reaction mixture was cooled to 0°C, followed by the addition of anhydrous AlCl_3_ (1.2 g, 9.0 mmol, 3 eq). The ice bath was allowed to melt naturally, and then raised to room temperature. A saturated aqueous NaHCO_3_ (20 mL) was then added to quench the reaction. The mixture was extracted with CH_2_Cl_2_ (3 × 50 mL). The combined organic layers were washed with CH_2_Cl_2_ (2 × 50 mL) and dried over anhydrous Na_2_SO_4_) The solvent was removed under reduced pressure, and the residue was purified by flash column chromatography (petroleum ether/EtOAc = 6:1) to afford the title product 2 as a colorless oil (yield 49.2%). ^1^H NMR (400 MHz, Chloroform-d) δ 6.89-6.80 (m, 2H), 6.80–6.71 (m, 3H), 5.99–5.91 (m, 4H), 5.84 (d, *J* = 9.1 Hz, 1H), 4.85 (d, *J* = 4.3 Hz, 1H), 4.26 (dd, *J* = 8.7, 7.7 Hz, 1H), 4.21–4.02 (m, 3H), 3.94 (t, *J* = 9.0 Hz, 1H), 3.11–2.96 (m, 1H), 2.08-1.98 (m, 7H).

#### 2.2.2 Synthesis of compound 3

To a stirred solution of compound 2 (1.0 g, 2.3 mmol) in a MeOH/H_2_O mixture (30 mL, MeOH/H_2_O = 10:1), K_2_CO_3_ (0.65 mg, 4.6 mmol) was added at room temperature. The reaction mixture was stirred for an additional 2 h. After completion of the reaction (checked by TLC), MeOH was evaporated under reduced pressure. The reaction mixture was extracted with ethyl acetate, and the combined organic layers were washed with brine (2 × 30 mL) and dried over Na_2_SO_4_. The crude product was purified by column chromatography on silica gel using ethyl acetate in petroleum ether to obtain the pure product as white solid in yield 90.20%. ^1^H NMR (400 MHz, Chloroform-*d*) δ 6.65-6.56 (m, 2H), 6.54-6.42 (m, 3H), 6.34 (s, 1H), 5.85 (dd, *J* = 19.8, 3.2 Hz, 4H), 4.03 (d, *J* = 13.1 Hz, 1H), 4.00–3.92 (m, 2H), 3.54-3.41 (m, 2H), 2.57 (td, *J* = 6.9, 2.4 Hz, 1H).

#### 2.2.3 Synthesis of compound JR20

Sodium bicarbonate (0.71 g, 8.46 mmol, 3 eq) was added to a stirred solution of compound **3** (1.0 g, 2.82 mmol) in acetonitrile (25 mL). The reaction mixture was cooled to 0°C, and Dess-Martin reagent (2.9 g, 6.8 mmol, 2.4 eq) was added slowly in batches. The reaction was stirred as the temperature rose from 0°C to room temperature and monitored by TLC. Completion was indicated by TLC after 2 h. The reaction mixture was poured into water and extracted with CH_2_Cl_2_ (3 × 20 mL). The organic layer was washed with brine, dried over Na_2_SO_4_, and evaporated. The residue was purified by column chromatography on silica gel, eluting with petroleum ether acetate (petroleum ether/EtOAc, 6:1) to yield JR20 as a yellow solid (yield: 72.3%). ^1^H NMR (400 MHz, CDCl_3_, *J* in Hz) δ (ppm): 9.69 (s, 1H), 9.49 (s, 1H), 7.45 (s, 1H), 6.85 (s, 1H), 6.71 (s, 1H), 6.65 (d, *J* = 8.00 Hz, 1H), 6.46 (dd, *J* = 8.16, 1.92 Hz, 1H), 6.41 (d, *J* = 1.84 Hz, 1H), 6.00 (s, 2H), 5.88 (dd, *J* = 3.04, 1.44 Hz, 2H), 4.69 (s, 1H), 3.94 (d, *J* = 1.84 Hz, 1H).

### 2.3 Antibacterial susceptibility testing

MRSA susceptibility to JR20 was assessed using the filter disc diffusion and microbroth dilution methods.

#### 2.3.1 Microbroth dilution assay

With only minor modifications, the procedure and criteria were adopted from the Clinical & Laboratory Standards Institute (CLSI) (Haley et al., 2024; Koeth et al., 2025). Three replicate wells per group were prepared and the bacterial suspension was diluted to 1–5 × 10^3^ CFU/mL (OD_600_ 0.030–0.060). Each 200 μL reaction contained 5 μL of test compound at graded concentrations plus 195 μL of the adjusted inoculum, the positive-control wells received VAN at a final concentration of 50 μg/mL. The negative-control wells received an equal volume of DMSO, and the blank consisted of sterile medium without bacteria. After 24 h incubation, the plate was read at 600 nm in a microplate reader. The inhibition rate was calculated as [1 – (OD_600_ Treated – OD_600_ Blank)/(OD_600_ Negative – OD_600_ Blank)] × 100 %. The seven-point concentration–response data were fitted with the variable-slope normalized response function in GraphPad to obtain the IC_50_.

#### 2.3.2 Filter disk diffusion assay

Discs of filter paper were punched into circles with a 6 mm diameter. A sterile cotton brush was used to evenly streak a bacterial suspension stimulated to 1–5 × 107 CFU/mL over agar plates. Using VAN as the positive control and DMSO as the negative control, JR20 was administered to the discs at doses of 2.5, 5, and 10 μg per disc. The discs were positioned with at least 20 mm between them on the infected agar surfaces. Following a 24-h incubation period at 37 °C, the inhibition zones were measured three times with a vernier caliper and interpreted using the following criteria: less than 10 mm indicates non-susceptibility, 11–15 mm indicates moderate susceptibility, 16–20 mm indicates high susceptibility, and more than 20 mm indicates very high susceptibility.

### 2.4 Evaluation of anti-biofilm effect

The anti-biofilm efficacy of JR20 was assessed using both crystal violet staining and dual-fluorescence labeling to evaluate its capacity to inhibit and disrupt fungal biofilm formation. Biofilms play a critical role in microbial colonization and represent a key mechanism contributing to drug resistance. These experimental approaches provide insight into the compound's potential in combating biofilm-associated antifungal resistance.

#### 2.4.1 Preliminary evaluation of biofilm inhibition effect

The MRSA bacterial suspension was adjusted to a concentration of 5 × 10^3^ CFU/mL and aliquoted into a 96-well plate. Compounds were added at concentrations corresponding to multiples of their respective IC_50_ values. Following 24-h incubation at 37 °C, the culture medium was carefully aspirated and the biofilm was fixed with methanol for 30 min. Crystal violet staining was performed for 30 min, after which the dye was removed. The plate was gently rinsed with PBS until the wash buffer became colorless. After air-drying overnight in an inverted position, the bound crystal violet was solubilized in anhydrous ethanol (Dmitrieva et al., 2022; Zhao et al., 2019). Absorbance was measured at 500 nm, and photographic documentation was obtained (Yang et al., 2023).

#### 2.4.2 Three-dimensional (3D) imaging of biofilm

Dual-color staining for biofilm viability was performed using the LIVE/DEAD™ BacLight™ Bacterial Viability Assay Kit. The fluorescent staining solution was prepared by mixing SYTO 9 and propidium iodide (PI) at the ratios specified in the kit instructions to achieve a final concentration of 6 μL/mL. MRSA bacterial suspensions (1–5 × 10^3^ CFU/mL) were seeded into two 8-well chamber slides with 500 μL per well. The wells were assigned to blank control, negative control, positive control, and drug-treatment groups, with concentrations set at multiples of the IC_50_. Slide 1 was designated for evaluating biofilm inhibition, while slide 2 was used to assess biofilm disruption. After allowing initial bacterial adhesion for 2 h, slide 1 was exposed to drug-containing medium and incubated for 24 h. Conversely, slide 2 was incubated for 24 h without treatment, then drug-containing medium was added, and incubation continued for an additional 24 h. Both slides were then gently rinsed with physiological saline, overlaid with 200 μL of the working solution, and stained in the dark for 30 min. After staining, coverslips were mounted, and three-dimensional images were acquired using dual fluorescence channels with FITC and PI excitation (Song et al., 2020).

Three-dimensional confocal microscopy images were processed using Fiji. Z-stacks were converted into two-dimensional representations via maximum intensity projection. A uniform segmentation threshold was applied consistently across all experimental groups. The projected total area (A _total_) was measured, along with the areas of specific fluorescent signals: red fluorescence (A _red_, indicating dead cells) and green fluorescence (A _green_, indicating viable cells). From these, the total fluorescent area (A _signal_ = A _red_ + A _green_) and the non-fluorescent background area (A _blank_ = A _total_ – A _signal_) were derived. The following proportional metrics were calculated:


$$\begin{array}{l} \text{Total fluorescence coverage }\left({\text{A}}_{\text{signal}}/{\text{A}}_{\text{total}}\times \;100\text{\%}\right)\\ \text{Death ratio }\left({\text{A}}_{\text{red}}/{\text{A}}_{\text{total}}\times \;100\text{\%}\right)\\ \text{Alive ratio }\left({\text{A}}_{\text{green}}/{\text{A}}_{\text{total}}\times \;100\text{\%}\right)\\ \text{Non}-\text{fluorescent area ratio}\left({\text{A}}_{\text{blank}}/{\text{A}}_{\text{total}}\times \;100\text{\%}\right) \end{array}$$

The proportional distribution of dead (red), alive (green), and blank (non-fluorescent) areas was visualized in a pie chart.

### 2.5 ATP content detection experiment

Intracellular ATP levels in MRSA were quantified using the CellTiter-Glo^®^ 2.0 luminescent assay (Promega Biotechnology, USA). The protocol was performed in strict accordance with the manufacturer's instructions, with minor adaptations to accommodate bacterial culture conditions. In this assay, ATP derived from metabolically active cells catalyzes a luciferase-based reaction, generating a luminescent signal proportional to the ATP concentration (Zhang et al., 2023).

We specifically selected this luminescence-based method over traditional colorimetric ATP assays because the inherent turbidity of dense bacterial suspensions can significantly interfere with optical density (OD) readings, leading to potential inaccuracies in absorbance-based quantification. The bioluminescent approach effectively circumvents this optical interference, providing a more reliable and specific measurement of ATP content within bacterial samples (Liang et al., 2022).

MRSA cultures in the logarithmic growth phase were plated into a 96-well plate at a concentration of 1–5 × 10^7^ CFU/mL at a volume of 100 μL per well. Different concentration gradients of JR20 were established alongside positive drug control VAN and negative DMSO control. All groups were incubated with the bacterial suspension for 24 h before removal. The prepared CellTiter-Glo^®^ 2.0 working solution was then added and the samples were lysed on ice for 15 min. Luminescence was detected using a multifunctional microplate reader (Synergy H1, California, USA) with an integration time of 0.25-1 s, and data were normalized by first subtracting the background and then setting the negative control to 100%. Results are presented as Relative Luminescence Units (RLU) on the y-axis.

### 2.6 Observation of ultramicroscopic surfaces and structures

#### 2.6.1 SEM characterization of bacterial morphology

To observe morphological changes, an MRSA suspension (1 × 107 CFU/mL) was treated with different concentrations of JR20, with VAN and DMSO serving as the positive and negative controls, respectively. The cultures were incubated at 37 °C and 150 rpm for 3 h. Following this, the samples were fixed overnight in 2.5% glutaraldehyde and subsequently dehydrated through a graded ethanol series (30%, 50%, 70%, 90%, and 100%), with 10-min incubations at each step. The ethanol was then replaced with isoamyl acetate, and the samples were freeze-dried for at least 1.5 h. Finally, the dried bacteria were mounted on a stub using conductive adhesive tape, sputter-coated with gold, and examined under a scanning electron microscope (GeminiSEM 300, UK).

#### 2.6.2 TEM characterization of bacterial internal structures

The activated bacterial suspension was adjusted to 1 × 10^7^ CFU/mL and treated with drug concentrations identical to those used in the SEM assay. After overnight fixation with a standard electron microscopy fixative, the bacterial cells were collected and embedded in 1% agarose. This was followed by secondary fixation with 1% osmium tetroxide for 2 h in the dark. The samples were then dehydrated through a graded ethanol series, following the same protocol as for SEM preparation but with acetone instead of isoamyl acetate as the final replacement solvent. Subsequently, the specimens were embedded in a 1:1 mixture of acetone and EPON 812 resin, polymerized at 60 °C for 48 h, and sectioned into ultra-thin slices. The sections were collected on carbon-coated copper grids, counter-stained, and finally observed under a transmission electron microscope (JEOL F200, Tokyo, Japan).

### 2.7 Molecular docking target prediction

The crystal structure of PBP2a (PDB: 1MWT), a key resistance determinant in MRSA, was obtained from the RCSB Protein Data Bank. The co-crystallized native ligand, penicillin G, was extracted for comparative analysis. The three-dimensional structure of JR20 was built using Chem3D (PerkinElmer), subjected to hydrogenation, and its torsion angles were optimized.

The protein was prepared by removing water molecules and adding polar hydrogens. Molecular docking was performed using AutoDock Vina (version 1.5.6) to assess the binding of JR20 and the reference ligand penicillin G to PBP2a. In each simulation, 1MWT was set as the receptor. A docking grid box of appropriate dimensions was centered on the active site, and ten independent docking runs were conducted. The conformation with the most favorable (lowest) binding energy was selected for analysis. The resulting complexes were visualized and analyzed using PyMOL (Schrödinger) and Discovery Studio 2021 (Dassault Systèmes).

### 2.8 JR20 anti-MRSA systemic infection study

#### 2.8.1 Animal experimental design and drug administration

A total of 60 male ICR mice (4 weeks old, 18–20 g) were randomly allocated into six experimental groups (*n* = 10 per group): blank control, negative control, positive control, and three JR20 treatment groups (low-, medium-, and high-dose). To establish the MRSA infection model, all mice except the blank control group received intraperitoneal injections of MRSA suspension (1 × 10^9^ CFU/mL) containing 10% mucin for two consecutive days.

#### 2.8.2 Drug formulation and treatment protocol

Following infection, each group received daily intraperitoneal injections of corresponding treatments for six consecutive days, with doses adjusted according to body weight. JR20 stock solutions were prepared at concentrations of 20, 40, and 60 mg/mL in DMSO and subsequently diluted tenfold with physiological saline to obtain working solutions containing 10% DMSO. Vancomycin hydrochloride (positive control) was processed similarly, with an initial stock solution of 40 mg/mL followed by tenfold dilution.

The treatment regimen included blank control receiving equivalent volume of physiological saline, negative control receiving physiological saline with DMSO concentration matching positive control formulations, positive control receiving vancomycin hydrochloride at 20 mg/kg/day, and JR20 treatment groups receiving low (10 mg/kg/day), medium (20 mg/kg/day), and high (30 mg/kg/day) doses.

#### 2.8.3 Tissue collection and processing

On day eight, following the 7-day treatment period, mice in these groups were all euthanized with CO_2_ at a flow rate of 30% chamber air displacement per minute (30% V/min; 1.2 L/min). This approach was specifically chosen to preserve tissue integrity and prevent potential interference of anesthetics with neutrophil activity and Ly6G epitope preservation, ensuring accurate assessment of neutrophil infiltration. Liver and spleen tissues were collected for hematoxylin and eosin (H&E) staining and Ly6G immunohistochemical staining to evaluate histopathological changes and therapeutic efficacy (Alalaiwe et al., 2018; Liu et al., 2021).

### 2.9 Data analysis

Statistical analysis was performed using GraphPad Prism 8.0 software (San Diego, California, USA). The experimental data were obtained from three independent replicates and visualized using line graphs and bar charts. Intergroup statistical differences were determined using the appropriate parametric or non-parametric tests.

*P* ≤ 0.05 was considered statistically significant. Significance levels are denoted as follows: ^*^ *p* ≤ 0.05, ^**^ *p* ≤ 0.01, ^***^ *p* ≤ 0.001, ^****^p ≤ 0.0001.

## 3 Results

### 3.1 Sythesis of arylnaphthalene lignan JR20

**SCHEME 1:**
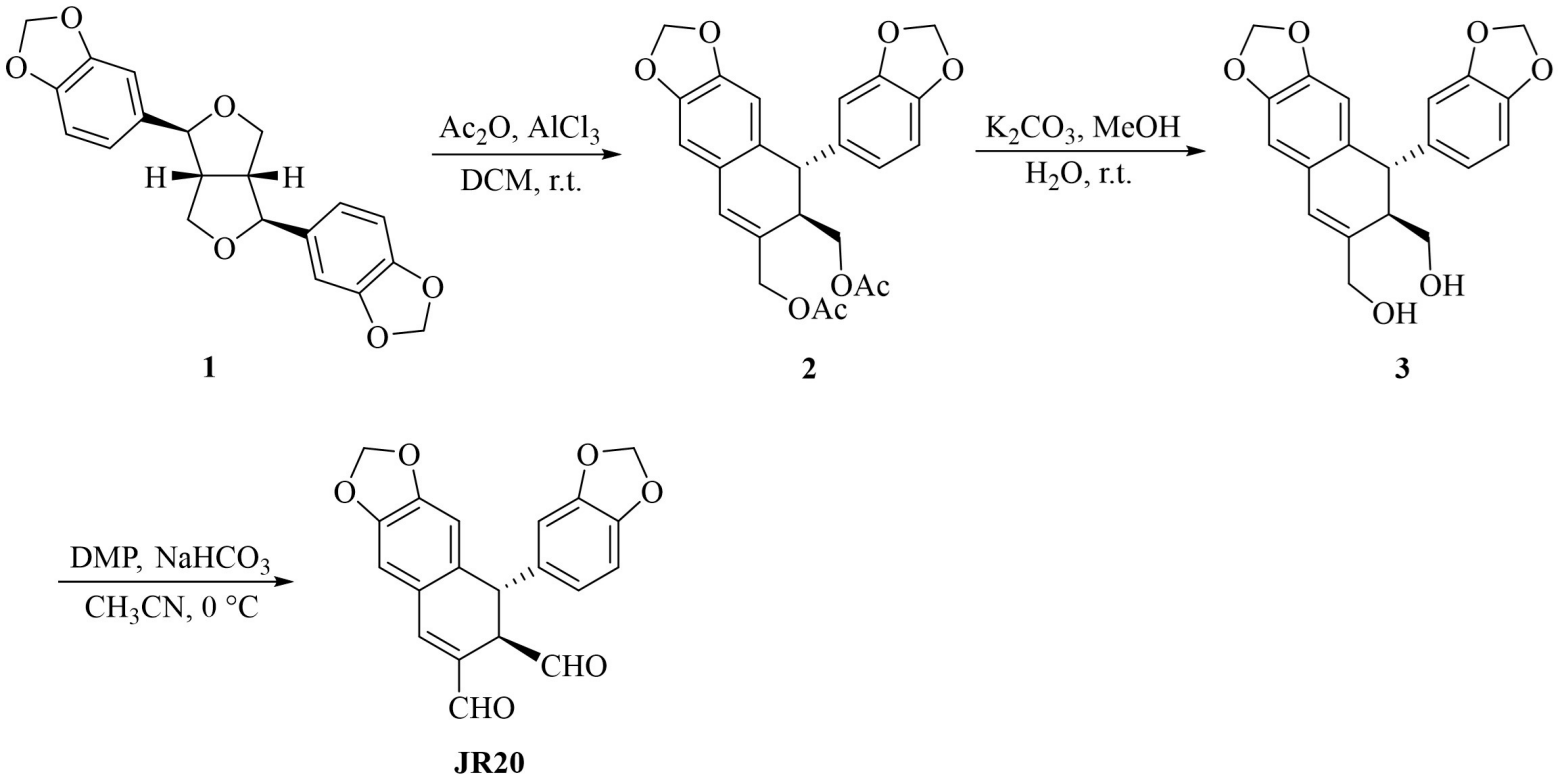
Synthetic route of JR20.

Our study revealed that the tetrahydrofuran ring in sesamin can be selectively cleaved to generate arylnaphthalene lignan analogs (Wu et al., 2024; Zhang et al., 2025). In the synthetic sequence, treatment of sesamin with AlCl_3_ induces ring opening of the tetrahydrofuran moiety, affording arylnaphthalene lignan 2. Subsequent deacetylation of compound 2 gives compound 3, which is further oxidized with Dess-Martin periodinane to afford the target compound JR20 (Scheme 1).

### 3.2 The antibacterial effect of JR20 on MRSA

**Figure 1:**
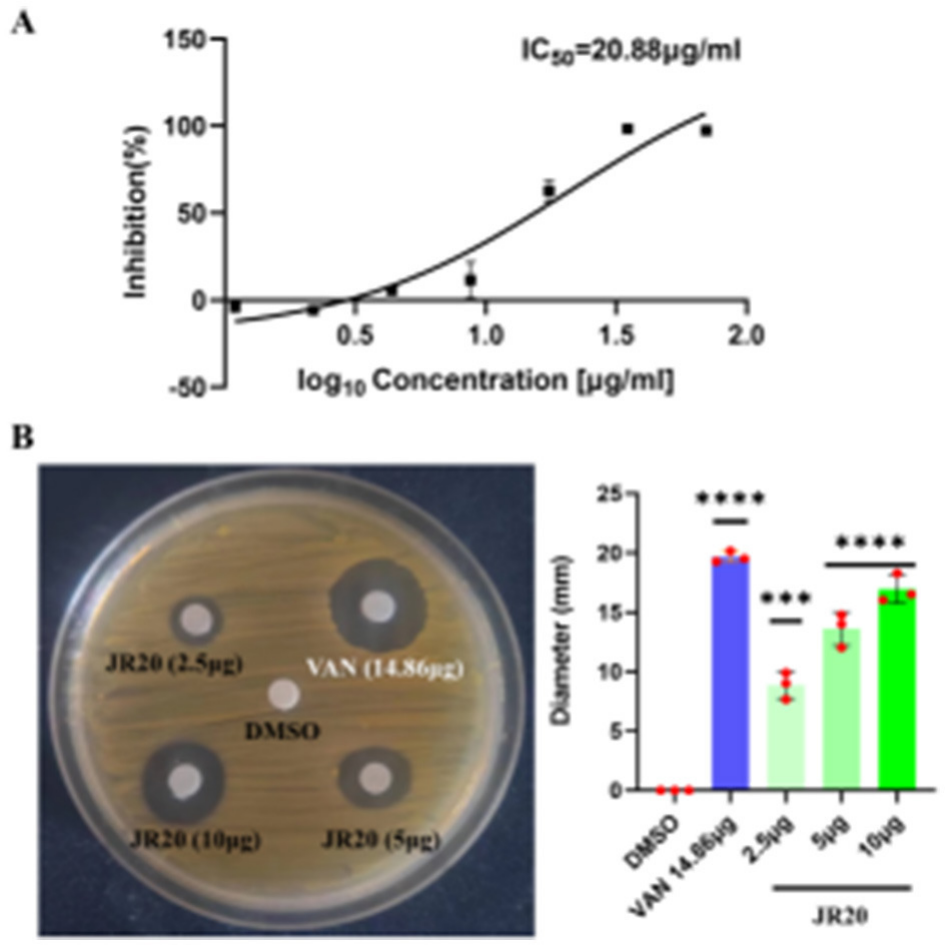
JR20′s anti MRSA activities in **(A)** IC50 value evaluation; **(B)** Antibacterial zone determination (*p < 0.5, **p < 0.01, ***p < 0.001, ****p < 0.0001 vs. DMSO control).

The anti-MRSA activity of JR20 was quantitatively and qualitatively evaluated through IC_50_ determination and disk diffusion assays. As shown in Figure 1A, JR20 exhibited an IC_50_ value of ~20 μg/mL against MRSA. In the agar diffusion test, distinct zones of inhibition were observed, with diameters positively correlated with drug concentration. At a dose of 10 μg, JR20 produced an average inhibition zone diameter of 16.9 mm, which was comparable to that of the vancomycin positive control (14.86 μg) and falls within the clinically defined highly sensitive range (Figure 1B). These results collectively demonstrate the potent *in vitro* anti-MRSA activity of JR20.

### 3.3 JR20 exhibits dual inhibitory and disruptive effects on MRSA biofilm

**Figure 2:**
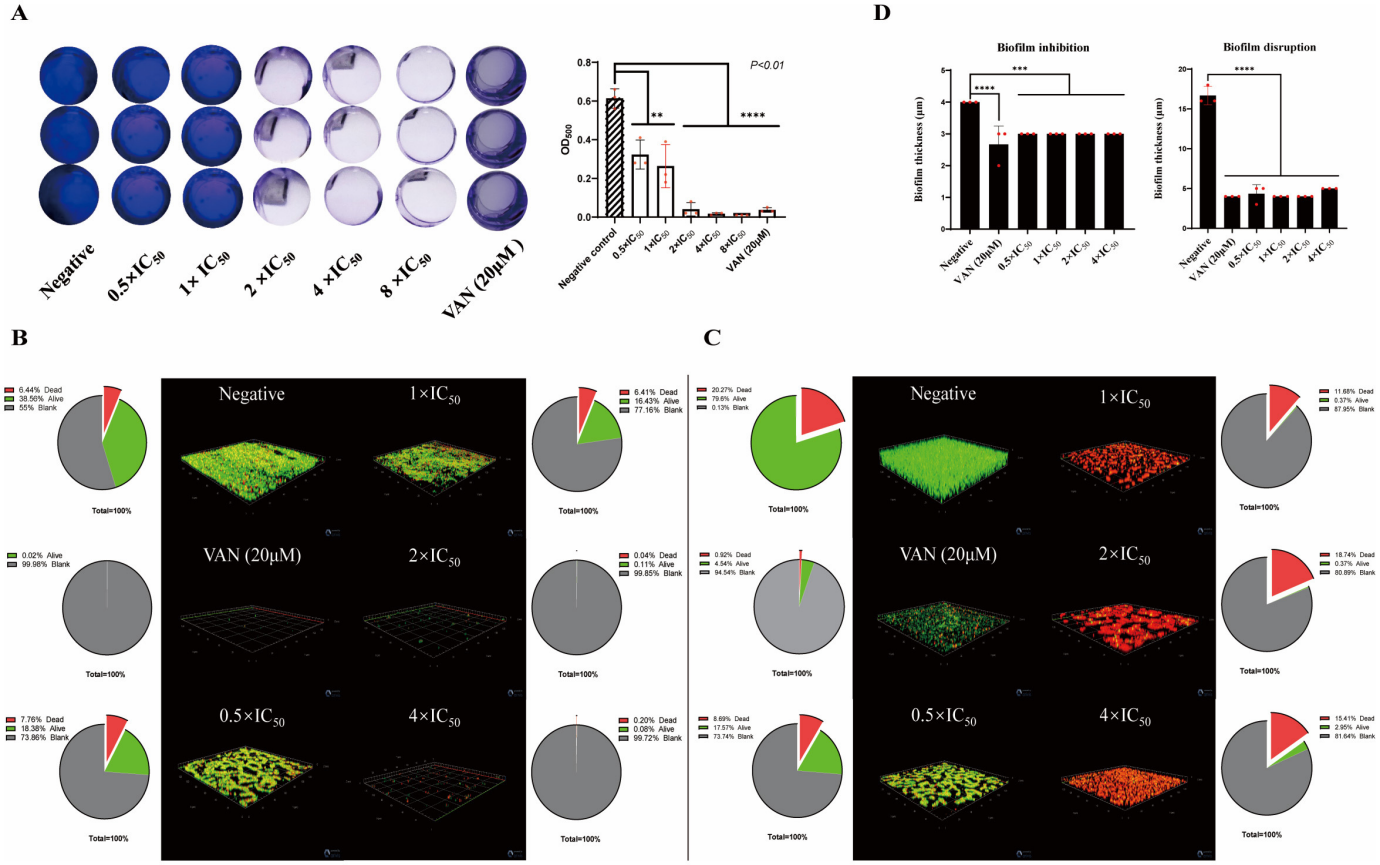
Effects of JR20 on MRSA biofilm formation and disruption. **(A)** Preliminary assessment of biofilm inhibition via crystal violet staining; **(B)** Biofilm inhibition activity and viability distribution (live/dead fluorescence ratios) under 3D confocal microscopy; **(C)** Biofilm disruption activity and viability distribution (live/dead fluorescence ratios) under 3D confocal microscopy; **(D)** Quantitative comparison of biofilm thickness following inhibition and disruption treatments (*p < 0.5, **p < 0.01, ***p < 0.001, ****p < 0.0001 vs. Negative control).

JR20 exhibited potent inhibitory effects on biofilm formation, as evidenced by the absence of crystal violet staining at concentrations approximating 2 × IC_50_, suggesting complete suppression of biofilm development (Figure 2A). In contrast, the compound's biofilm disruption capacity was not clearly demonstrated in this initial assay.

SYTO 9 dye typically labels all bacteria within a population—regardless of whether their cell membranes are intact. PI, however, can only penetrate bacteria with damaged membranes. Upon binding to DNA, PI exhibits enhanced fluorescence intensity, displacing SYTO 9 and causing dead cells to appear red instead of green (Stiefel et al., 2015). Thus, bacteria with intact cell membranes exhibit green fluorescence, while those with damaged membranes appear red. The excitation/emission peaks for these dyes are: SYTO 9 at approximately 480/500 nm, and PI at ~490/635 nm (Mcgoverin et al., 2020).

Confocal microscopy was employed to quantitatively assess the inhibitory and disruptive effects of JR20 against MRSA biofilms, using red/green fluorescence intensity and biofilm thickness as key metrics, over a total imaging area of 135 × 135 μm^2^. In the biofilm inhibition assay (Slide 1, Figure 2B), both the positive control vancomycin (VAN) and JR20 at 2 × IC_50_ and 4 × IC_50_ effectively suppressed biofilm formation, as reflected by negligible fluorescence signals (non-fluorescent area > 99 %) and minimal thickness (~3 μm) (Figure 2D). At lower concentrations (0.5 × IC_50_ and 1 × IC_50_), moderate inhibition was observed, with biofilm formation rates of only 16–18 % relative to the negative control.

In the biofilm disruption assay (Slide 2, Figure 2C), JR20 at 0.5 × IC_50_ induced partial bacterial death (8.69 %) and reduced biofilm thickness. Higher concentrations (1 × IC_50_ to 4 × IC_50_) progressively enhanced damage, marked by further decreases in thickness, a gradual rise in the red-fluorescence ratio from 11.68% to 15.41%, and nearly absent green fluorescence, consistent with widespread loss of membrane integrity. Of note, the total fluorescent area slightly exceeded the sum of the individual red and green areas, indicating the presence of mixed signals or non-specific background fluorescence—an expected feature of this experimental system. The accompanying pie chart illustrates the relative proportions of red (dead cells), green (live cells), and non-fluorescent areas within the total projected area; these values represent normalized distributions, as the measured regional areas did not sum to 100% due to signal overlap and background noise.

Collectively, these concentration-dependent effects demonstrate JR20′s dual capacity to inhibit biofilm formation and disrupt pre-formed biofilms, supporting further investigation into its underlying mechanisms and suggesting potential bactericidal activity against MRSA.

### 3.4 Ultrastructural alterations in MRSA cell envelope

**Figure 3:**
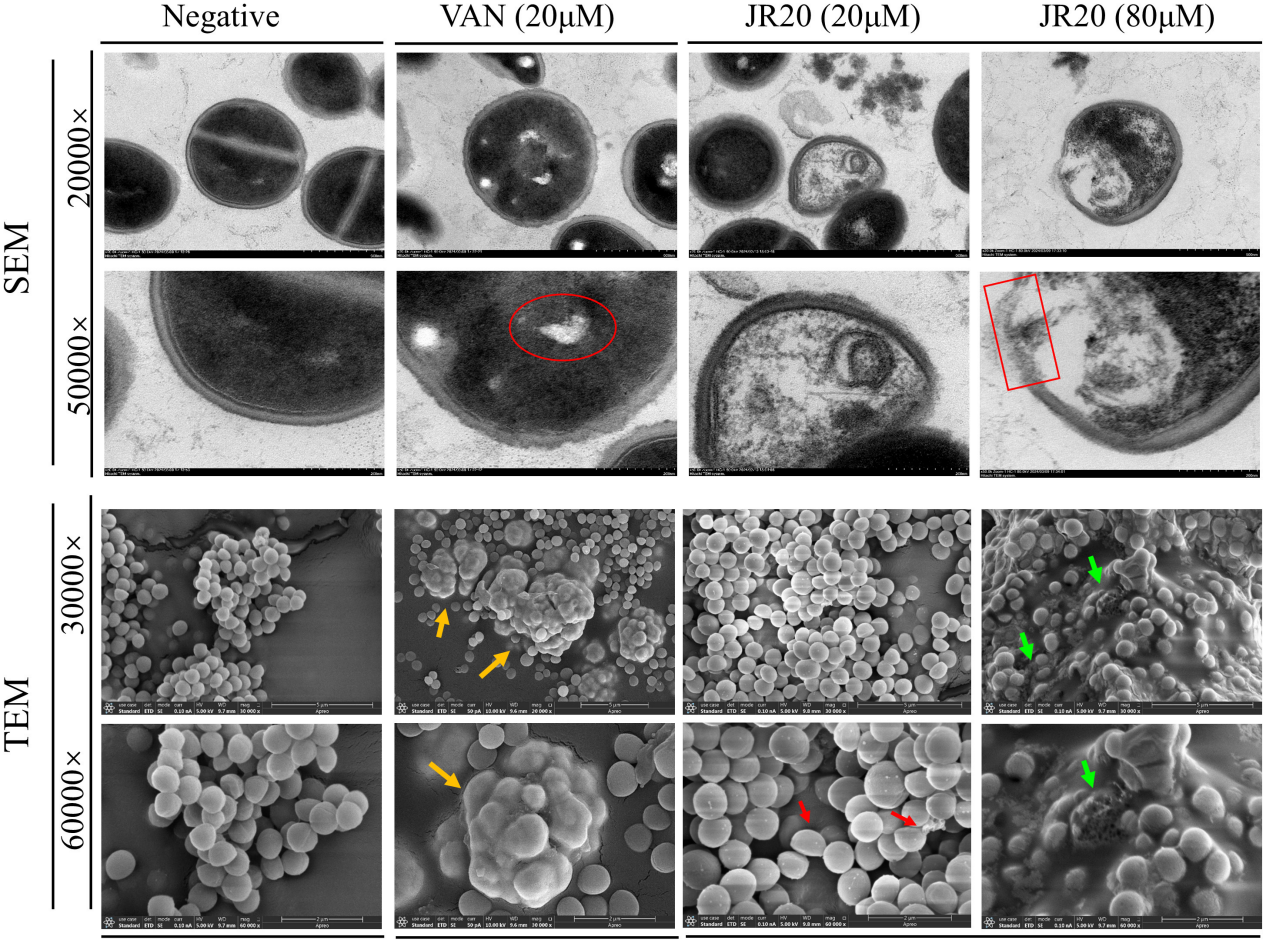
Morphological alterations of MRSA induced by JR20 revealed through scanning and transmission electron microscopy (Red circle: cytolysis; Yellow arrows: biofilm proliferation; Red arrows: cellular collapse and minor cytoplasmic leakage; Red box: cell wall damage; Green arrows: biofilm disruption).

Figure 3 presents scanning and transmission electron microscopy images of MRSA following treatment. In the negative control group, MRSA cells displayed typical morphology with clear division septa, intact cell walls, smooth surfaces, and uniformly turgid cellular structures. In the vancomycin (VAN) positive control group, early signs of cytoplasmic dissolution were observed (red circle), accompanied by ill-defined cellular boundaries and tumor-like aggregated structures under SEM, along with visible biofilm proliferation (yellow arrows).

Treatment with low concentrations of JR20 resulted in cellular collapse and minor cytoplasmic leakage (red arrows). At higher JR20 concentrations, significant tumor-like aggregation, extensive biofilm-like material or cellular debris accumulation (green arrows), and substantial cell wall damage (red box) were evident. These morphological alterations demonstrate that JR20 disrupts MRSA cell wall integrity and interferes with biofilm formation, ultimately leading to bacterial necrosis.

### 3.5 JR20 causes metabolic collapse in MRSA

**Figure 4:**
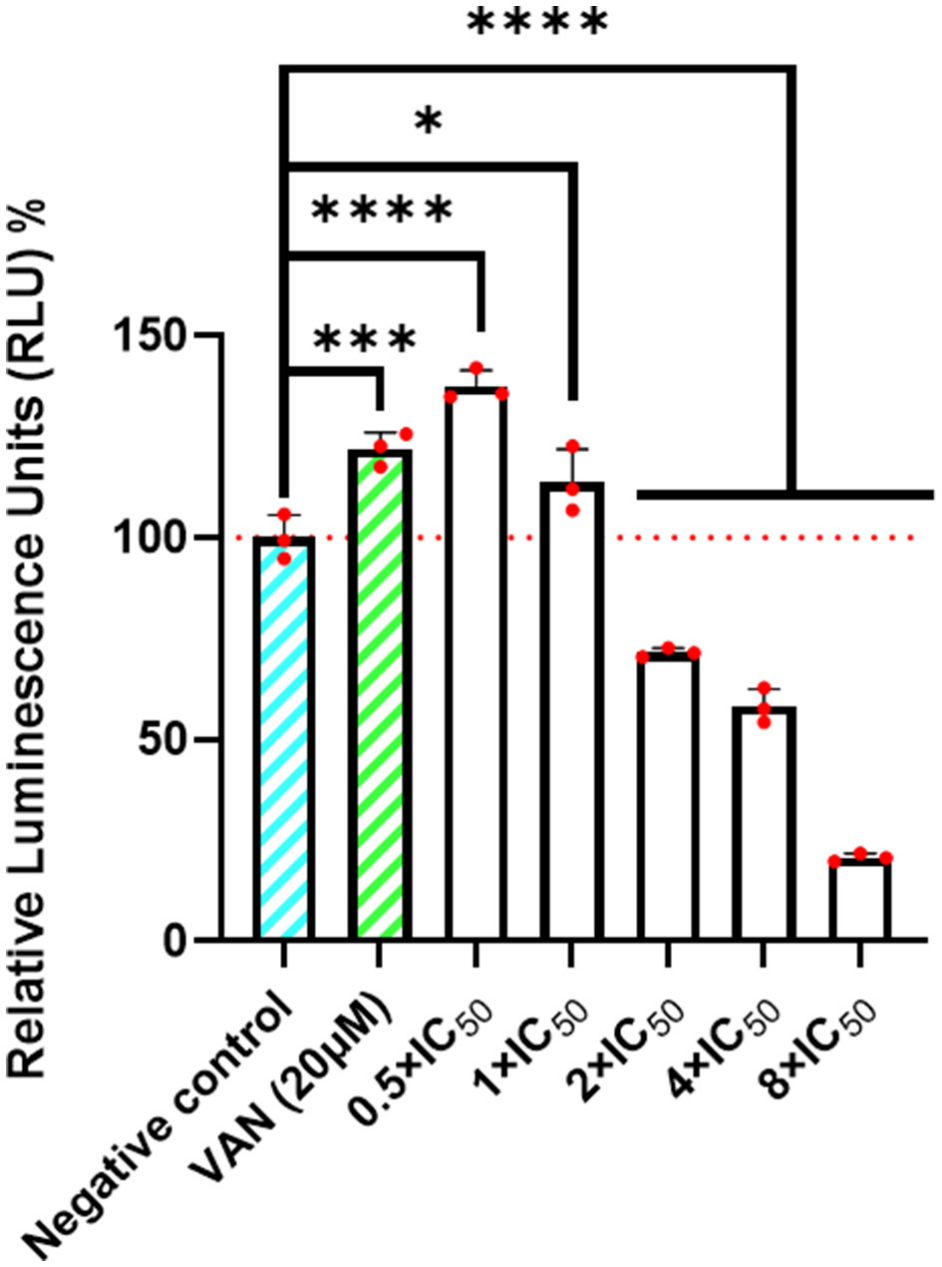
JR20 reduces ATP levels in MRSA, suggesting its antibacterial activity may be associated with pro-necrotic effects (*p < 0.5, **p < 0.01, ***p < 0.001, ****p < 0.0001 vs. Negative control).

Intracellular ATP levels serve as a key indicator of antibacterial efficacy and provide insights into the drug's mechanism of action. As shown in Figure 4, at low concentrations of JR20 (1 × IC_50_ to 2 × IC_50_), ATP levels initially increased relative to the negative control, a trend also observed with 20 μM vancomycin (VAN). This transient rise suggests that MRSA may activate a stress response to counteract the drug by boosting metabolic activity. However, as the JR20 concentration increased from 2 × IC_50_ to 8 × IC_50_, ATP levels progressively declined, indicating a shift from transient metabolic activation toward metabolic collapse and cell death. The marked ATP suppression at elevated concentrations likely reflects irreversible cellular damage, potentially mediated through cell wall disruption or inhibition of key enzymatic activities. This concentration-dependent ATP depletion correlates well with the ultrastructural damage visualized by scanning and transmission electron microscopy (SEM/TEM), collectively supporting the conclusion that JR20 induces bacterial necrosis. These converging lines of evidence underscore the need for further investigation into the precise mechanism of action of JR20.

### 3.6 JR20 forms hydrogen bonds with PBP2a

**Figure 5:**
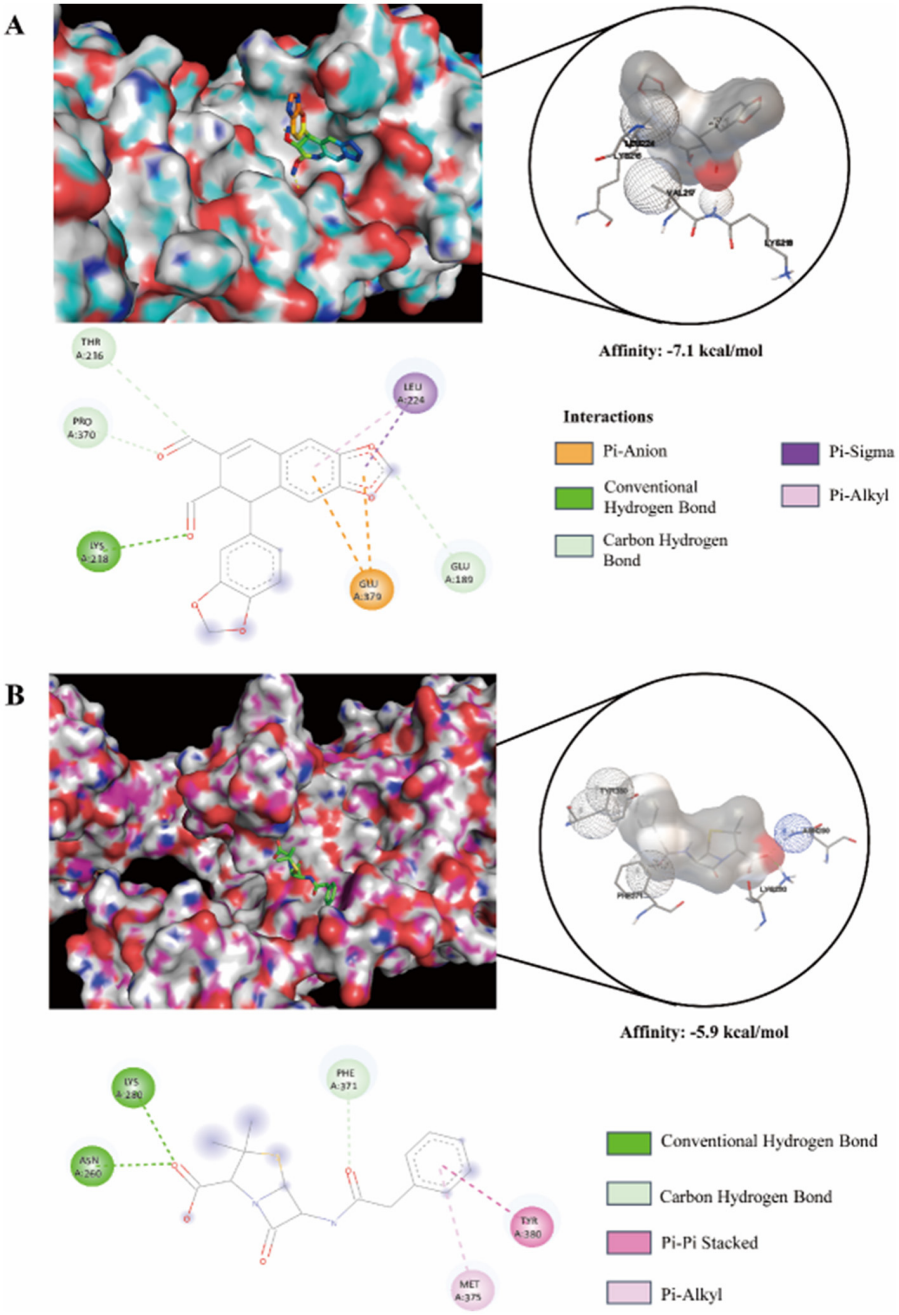
The results of molecular docking and affinity between protein and small molecule **(A)** JR20 and **(B)** Penicillin G with PBP2a.

Molecular docking analysis was performed to gain preliminary mechanistic insights into the JR20-PBP2a interaction. Analysis using visualization software (PyMOL and Discovery Studio 2021) indicated that JR20 binds to PBP2a with a high affinity (−7.1 kcal/mol, Figure 5A), which is stronger than that of penicillin G (−5.9 kcal/mol, Figure 5B). Structural observations further revealed that JR20 forms one hydrogen bond with Lys218 and additional carbon-hydrogen bonds with neighboring residues within the non-binding allosteric domain. These interactions appear to collectively contribute to complex stability. Taken together, these results suggest that JR20 may modulate PBP2a function via binding to this allosteric domain.

Previous studies indicate that the α2-α3 and β3-β4 loop regions in PBP2a, shield the active site and impede antibiotic entry into the active pocket. Conformational changes in the β3 chain and the N-terminal region of the α2 helix are essential for β-lactam antibiotics to approach Ser403 (Mahasenan et al., 2017; Rosado et al., 2025). Based on these findings, we propose a bold hypothesis: although JR20 does not occupy the active site of this protein, its binding position suggests a possible allosteric regulatory mechanism. Namely, JR20 binds to the active region within the enzyme's N-terminal non-penicillin-binding domain, inducing an allosteric effect that inhibits cell wall synthesis.

### 3.7 Evaluation of JR20′s efficacy against MRSA systemic infections

Ly6G, as a neutrophil-specific marker, is commonly used in immunohistochemical (IHC) or immunofluorescence (IF) staining analyses. In H&E staining, cell nuclei appear vivid blue after hematoxylin staining and mucus appears grayish-blue. Cytoplasm exhibits a pink-to-peach gradient after eosin staining, and intracellular eosinophilic granules show bright red reflectivity (Adrover et al., 2025; Feldman and Wolfe, 2014).

**Figure 6:**
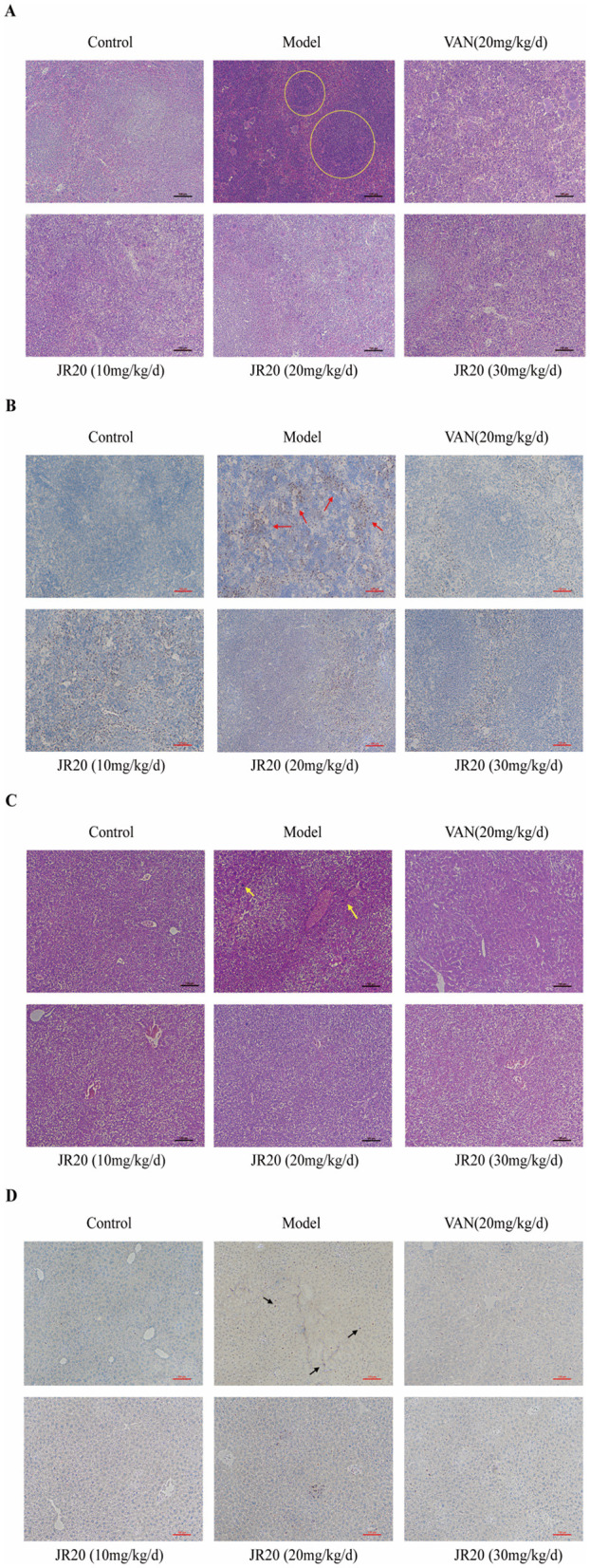
JR0′s efficacy against MRSA infections in mouse liver and spleen under JR20 treatment **(A)** H&E staining results of mouse spleen; **(B)** Ly6G staining results of mouse spleen; **(C)** H&E staining results of mouse liver; **(D)** Ly6G staining results of mouse liver (100×, scale bar = 100 μm; Yellow circles: inflammatory infiltration; Red arrows: spleen neutrophil infiltration; Yellow arrows: substantial fibrosis; Black arrows: liver neutrophil infiltration).

Spleen H&E staining revealed: the model group exhibited extensive deep blue inflammatory infiltration with blurred splenic corpuscle borders and prominent red eosinophilic granules. This indicated the activation of inflammation-mediated immune responses (yellow circles). This feature was significantly improved in the JR20-treated group (Figure 6A). Ly6G staining revealed extensive darkly stained neutrophil infiltration around splenic corpuscles in the model group (red arrows). Partial neutrophil infiltration persisted in the low-dose JR20 group, while the high-dose group showed markedly reduced infiltration (Figure 6B).

Liver H&E staining demonstrated fibrotic necrosis in the hepatic parenchyma of the model group (yellow arrows). Fibrotic lesions remained present in the low-dose JR20 group. Whereas, no significant abnormalities were observed in the high-dose group (Figure 6C). Ly6G staining similarly revealed marked neutrophil infiltration in the model group liver (black arrows), with reduced infiltration observed across all treatment groups (Figure 6D). These sections indicate that extensive neutrophil infiltration and inflammatory infiltration characteristics in the model group, collectively suggest a state of profound inflammation. JR20 effectively alleviates this pathological manifestation, with therapeutic effects exhibiting a concentration-dependent pattern.

## 4 Conclusion

This study comprehensively evaluated the anti-MRSA activity of JR20 (IC_50_ = 20.88 μg/mL) through both *in vitro* and *in vivo* models. *In vitro* analyses revealed its dual mechanism of action: at low concentrations, JR20 triggered a bacterial stress response, characterized by increased ATP levels, cellular collapse, and early cytoplasmic dissolution; At high concentrations, it caused cell wall disruption, cytoplasmic leakage, and ATP depletion. Molecular docking predicted an interaction between JR20 and the key resistance-related protein PBP2a, with a binding affinity of −7.1 kcal/mol, offering a preliminary mechanistic insight.

*In vivo* efficacy was confirmed in a murine model, where histological (H&E) and immunohistochemical (Ly6G) staining of liver and spleen tissues demonstrated that JR20 significantly reduced inflammatory stress and neutrophil infiltration.

Previous cytotoxicity assessments reported CC_50_ values of 14.48 μM for HEK 293T cells and 13.03 μM for RAW 264.7 cells (Zhang et al., 2025), indicating non-negligible cytotoxicity. Despite this limitation, JR0′s specific and potent anti-biofilm activity establishes it as a promising lead compound. Its structure holds significant potential for further chemical modification and optimization aimed at reducing toxicity while retaining or enhancing its unique antimicrobial properties.

A major highlight of this study lies in the use of confocal microscopy combined with dual-fluorescent staining to achieve three-dimensional visualization of JR0′s action on MRSA biofilms. The images demonstrate its concentration-dependent inhibitory and disruptive effects on biofilms, showing strong correlation with theoretical membrane thickness and fluorescence ratio results. JR0′s exceptional anti-biofilm properties against MRSA demonstrate its significant potential as a natural product derivative for developing anti-drug-resistant bacterial therapeutics. Although some cytotoxicity was observed, its multifaceted antibacterial mechanisms warrant further investigation through structural modification studies. Subsequent work will focus on structural optimization and mechanism of action elucidation for JR20, providing new insights for developing natural product-based therapies against drug-resistant bacterial infections.

## 5 Discussion

JR20 exhibits broad-spectrum antibacterial activity and favorable biological properties, though its cytotoxicity profile warrants further evaluation as previously reported. Although this study did not fully elucidate JR0′s mechanism of action against MRSA, we conducted molecular docking analysis targeting PBP2a—a characteristic resistance factor in MRSA. Results indicate that, although JR20 does not directly bind to the active site or its adjacent regions, its behavior reveals a key strategy for countering β-lactam resistance: enhancing the antimicrobial efficacy of weak PBP2a inhibitors through the use of adjuvants (Islam et al., 2024). This approach aims to develop adjuvant drugs that specifically target allosteric sites of PBP2a, forcing the active site to open toward the catalytic inhibitor and thereby inhibiting protein function. This is considered one of the most promising directions for developing novel anti-MRSA drug (Witek et al., 2023).

Cephalosporins represent a subclass of β-lactam antibiotics introduced in the 1960s. Among them, ceftaroline, as a fifth-generation cephalosporin, exhibits higher affinity for the PBP2a protein than other β-lactam antibiotics. Research indicates that binding of ceftaroline to the allosteric site of PBP2a induces conformational changes, particularly significant displacement in the β3-β4 loop region near the active site, causing ~10 Å movement of the Cα atoms of Arg612 and Gln607 (Guignard et al., 2005). Another example is the 1-benzhydrylpiperazine-5-spirofluorenehydantoin derivative, which enhances oxacillin activity by binding to the PBP2a allosteric site. At a concentration of just 62.5 nM, it restored antimicrobial efficacy against six out of eight MRSA strains (Witek et al., 2023). Furthermore, when active natural products were combined with β-lactam antibiotics, the latter exhibited significantly reduced IC_50_ values. This synergistic effect similarly indicates that natural product-based adjuvants possess the potential to synergize with PBP2a and inhibit MRSA activity. Future research on JR20 will integrate the electron microscopy and molecular docking results from this study to further elucidate its mechanism of action.

Biofilms are key virulence factors of MRSA, directly driving its infection *in vivo*. JR20 effectively disrupts MRSA biofilms, highlighting its unique potential against drug-resistant bacteria. This capability is significant for countering complex biofilm resistance mechanisms, and establishing its value as a key candidate compound. Additionally, JR20 exhibits dual antifungal and antibacterial activity, distinguishing it from conventional drugs and positioning it as a potential lead compound for novel antimicrobial development. This opens new perspectives for natural product-based strategies against drug resistance.

## Data Availability

The original contributions presented in the study are included in the article/Supplementary material, further inquiries can be directed to the corresponding authors.
