# Supplementary material for: JR20, a novel natural product-derived compound, exhibits potent anti-biofilm activity against methicillin-resistant Staphylococcus aureus
Source: Front Microbiol. 2026 Jan 22;16:1743534. doi: 10.3389/fmicb.2025.1743534 (PMC12872765; doi:10.3389/fmicb.2025.1743534)
Supplement: Supplementary file 1 [file Data_Sheet_1.docx]

**JR20, a Novel Natural Product-Derived Compound, Exhibits Potent Anti-Biofilm Activity Against Methicillin-Resistant *Staphylococcus aureus***

**Meirong Zhao^1,2†^, Chaowei Zhang^3,4†^, Yogini Jaiswal^5^, Xinrong Xie^1^, Dongyu Huang^6^, Zhendan He^3^, Leonard Williams^5^, Yifu Guan^1*^, Hedong Bian^1*^, Xun Song^3*^**

**^1^**School of Chemistry and Chemical Engineering, Guangxi Minzu University, Nanning, 530006, China; [zhaomeirong124@163.com](mailto:zhaomeirong124@163.com) (M.Z.)

**^2^**College of Food and Pharmaceutical Engineering, Guangxi Vocational University of Agriculture, Nanning 530006, China;

**^3^**College of Pharmacy, Shenzhen Technology University, Shenzhen, 518118, China; [hezhendan@sztu.edu.cn](mailto:hezhendan@sztu.edu.cn) (Z.H.)

**^4^**School of Chinese Medicine, Hong Kong Baptist University, Kowloon Tong, Hong Kong, China; [25481797@life.hkbu.edu.hk](mailto:25481797@life.hkbu.edu.hk) (C.Z.)

**^5^**Center for Excellence in Post-Harvest Technologies, North Carolina Agricultural and Technical State University, The North Carolina Research Campus, Kannapolis, NC 28081, USA

**^6^**School of Pharmacy, Shenzhen University Medical School, Shenzhen University, Shenzhen, 518000, China; [18218793343@163.com](mailto:18218793343@163.com) (D.H.)

*****Correspondence: [guanyifu@gxmzu.edu.cn](mailto:guanyifu@gxmzu.edu.cn) (Y.G.); [bianhd@gxmzu.edu.cn](mailto:bianhd@gxmzu.edu.cn) (H.B.); songxun@sztu.edu.cn (X.S.);

**^†^** These authors contributed equally to this work.

Supplementary Material

# Supplementary Figures
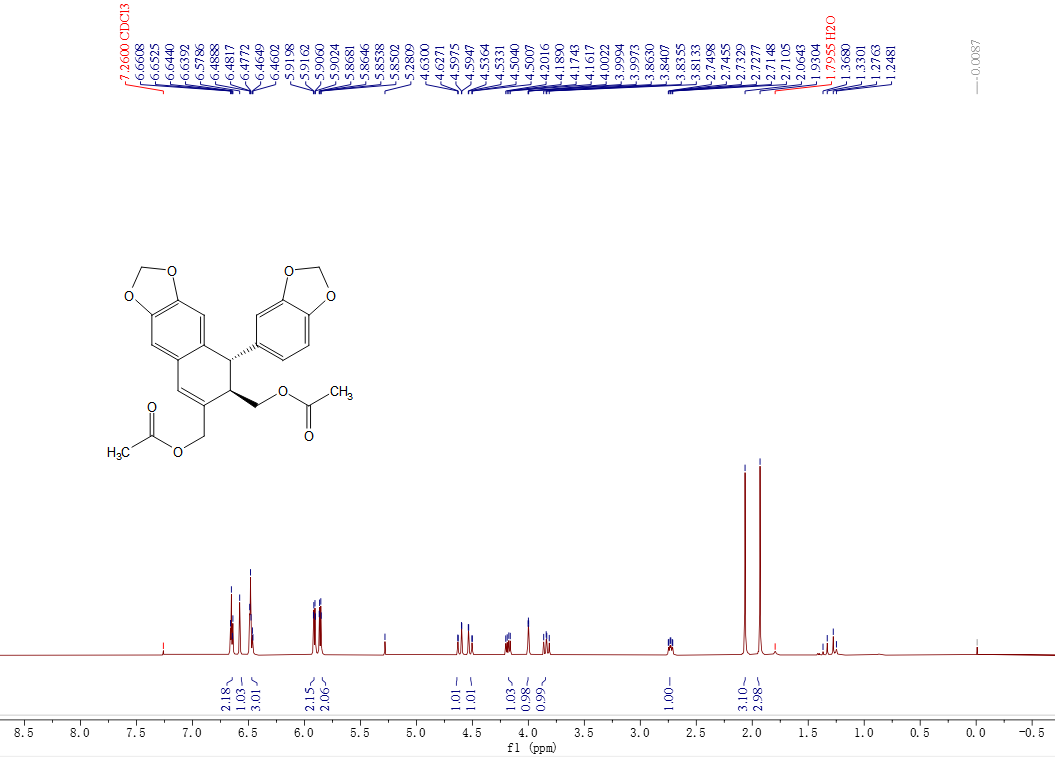


**Figure S****1.** The ^1^H NMR spectrum of compound **2** in CDCl_3_ (400 MHz)


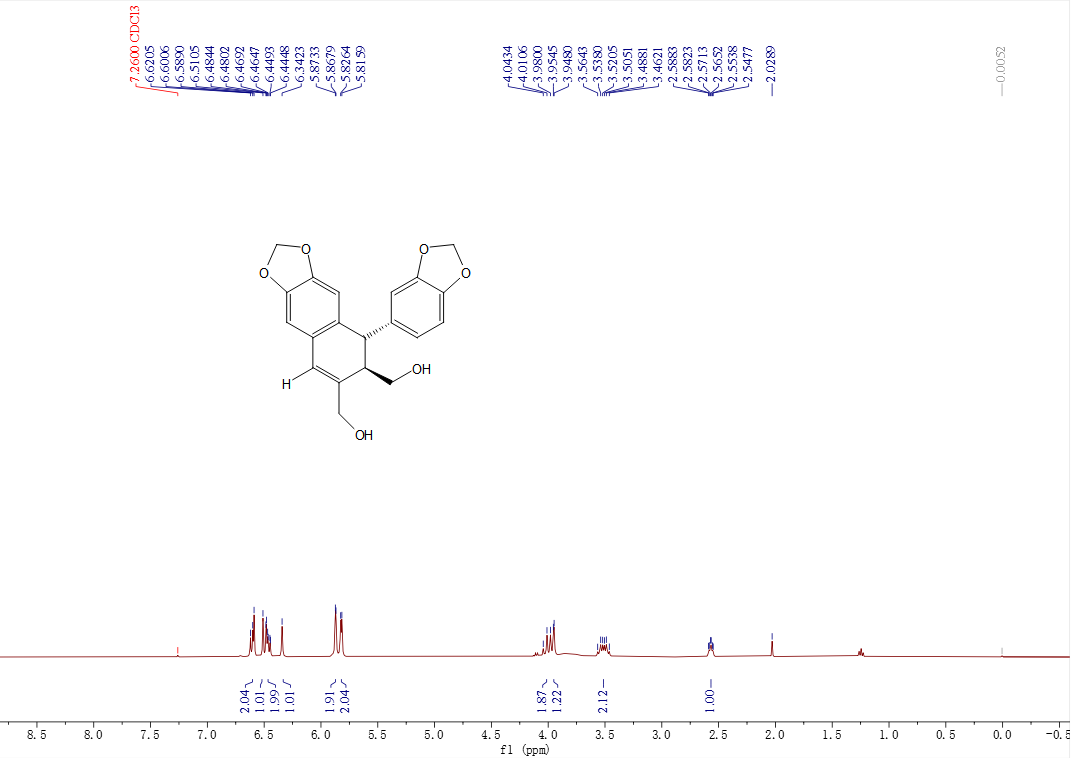


**Figure S2.** The ^1^H NMR spectrum of compound **3** in CDCl_3_(400 MHz)


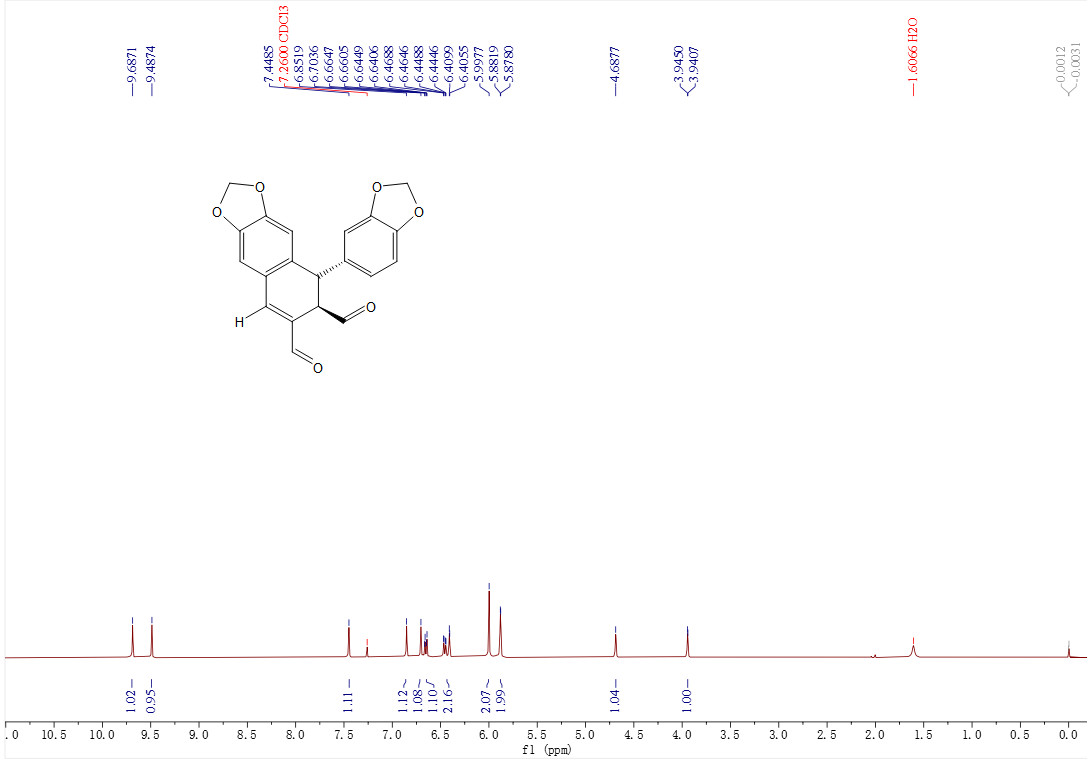


**Figure S3.** The ^1^H NMR spectrum of compound **JR20** in CDCl_3_ (400 MHz)
